# An evaluation of written materials for supporting hypertensive patient education and counselling when performing a new medicine service in Poland

**DOI:** 10.1186/s12909-024-05523-x

**Published:** 2024-05-10

**Authors:** Magdalena Jasińska-Stroschein, Justyna Dymek, Mariola Drozd, Olga Sierpniowska, Artur Jędra, Agnieszka Stankiewicz, Paulina Stasiak, Sylwia Cholewa, Magdalena Nowakowska, Magdalena Waszyk-Nowaczyk

**Affiliations:** 1 Department of Biopharmacy, Medical University of Lodz, Muszynskiego 1, Lodz, 90-151 Poland; 2 Department of Social Pharmacy, Jagiellonian University Medical College, Medyczna 9, Kraków, 30-688 Poland; 3 Department of Humanities and Social Medicine, Medical University of Lublin, Al. Racławickie 1, Lublin, 20-059 Poland; 4 Hospital Pharmacy, Independent Public Health Care Institution in Szczebrzeszyn, Zygmunta Klukowskiego 3, Szczebrzeszyn, 22-460 Poland; 5 Council of District Chamber of Pharmacy, Zeromskiego 77/6, Warsaw, 01-882 Poland; 6 Lubusz Pharmacy Chamber Council, Wojska Polskiego 37, Pharmacy, Zielona Góra, 65-077 Poland; 7 Pharmacy Practice and Pharmaceutical Care Division, Department of Pharmaceutical Technology, Poznan University of Medical Sciences, Grunwaldzka 6, Poznan, 60-780 Poland

**Keywords:** Anti-hypertensive therapy, Medication adherence, Pharmaceutical care, New medicine service, Written educational material

## Abstract

Background. The New Medicine Service (NMS) was developed in England more than ten years ago, as a three-stage consultation led by community pharmacists to support patients taking new medication for a chronic disease. In Poland, the scheme was officially introduced in January 2023. However, its implementation into common practice has been presented with various obstacles, including the need to develop relationships with general practitioners, resolve the payment structure, and provide training with adequate supporting materials. Hence, written materials have been designed for use as an optional tool for counselling patients receiving an NMS in community pharmacies. Methods. The present study evaluates the ability of these materials to inform patients about the need to adhere to anti-hypertensive medication. A group of 401 randomly-selected adult visitors to pharmacies and/or healthcare centres were surveyed; one third had hypertension in their history. Results. The structure, grammar and readability of the text achieved the required threshold of 40% according to the Plain Language Index. The designed materials effectively informed the patients about anti-hypertensive medication, reflected in an increased score in a knowledge test, and were rated positively regarding information level, comprehensibility and presentation. Conclusion. The proposed material may serve as an additional, “patient-friendly” educational tool for use as part of an NMS.

**Supplementary Information:**

The online version contains supplementary material available at 10.1186/s12909-024-05523-x.

## Background

**Fig. 1:**
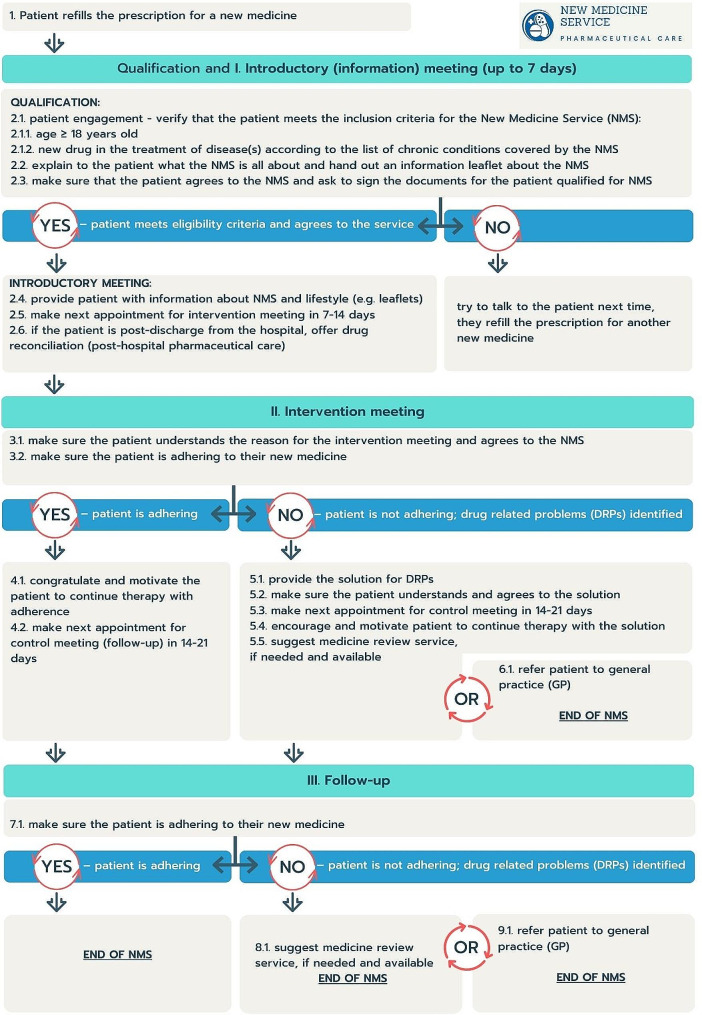
New medicine service flow chart – Polish adaptation

The New Medicine Service (NMS) was introduced in England in 2011 by community pharmacists to support patients taking new medication for a chronic disease. Briefly, the NMS consists of a structured and documented one-to-one intervention comprising a set of three consultations, to be completed within the first four weeks of starting a new medication; its aim is to resolve individual patient specific drug problems by providing information, education and advice [1]. Recent findings indicate that the NMS offers various potential benefits, including supporting better medication adherence for chronically-ill patients, including these starting on cardiovascular medication [2, 3]. Its educational function covers the supplementation and reinforcement of information provided by the prescriber, to help patients make informed choices about their care. An NMS was officially introduced in Poland in January 2023 by the present authors, as a three-stage, structured consultation supported by a set of proposed documents (Fig. 1) [4, 5]. The introduction was made possible by legislation in 2020 obliging pharmacists to provide pharmaceutical care. Pharmacists recently were presented with the opportunity to take a more patient-centred role beyond simply dispensing medicines by performing standardized pharmaceutical consultations (e.g., NMS), medication reviews, prescribing the fully-reimbursed drugs *pro auctore* and *pro familiae*, and administering COVID-19 vaccinations; however, the service faces many problems, particularly lack of financing. Nevertheless, cardiovascular disease (CVD) with arterial hypertension (HTN) is currently eligible for pharmaceutical consultation, including NMS.

CVD remains a predominant cause of illness and death globally [6]. Poor control of HTN has been correlated with a higher risk of cardiac abnormalities including myocardial infarction, stroke, ischemic and brain haemorrhage and peripheral arterial disease [7]. The prevalence of HTN is believed to be 32.5% among Polish residents between 18 and 80 years of age, and that among 10.8 million Polish patients suffering from HTN, only 2.6 million are receiving successful treatment. Current guidelines indicate one major cause of poor control of BP is non-adherence to treatment [8, 9].

A variety of interventions aimed at improving medication adherence in HTN have been assessed to date. These approaches include patient education about medication therapies (e.g., their rationale, proven benefits, indications, contraindications, adverse reactions, dosage regimens), counselling about secondary lifestyle modifications (e.g., blood pressure control, alcohol and smoking cessation, exercise, healthy diet, etc.), as well as monitoring and risk screening [10]. Other tools could include adherence aids (pillbox which the patient practices filling, personalized illustrated medication schedule), event diaries (e.g., symptom monitoring diary cards) as well as educational booklets and leaflets (written, audiovisual or downloadable formats) [11]. Some of these tasks might be completed with different types of eHealth tools, including phone calls, blood pressure telemonitoring, emails, websites, smartphone applications and SMS [12].

The current paper evaluates the ability of a set of written educational materials to inform patients regarding adherence to anti-hypertensive medications. The analysis was performed on material introducing angiotensin converting enzyme inhibitors (ACEI) and thiazide/thiazide-like diuretics, the most popular first-line anti-hypertensive class agents in Poland. The materials, in Polish and English language versions, were designed to be downloaded from the national website for the NMS [13].

## Methods

### Study design and participants

The study was performed as an observational survey with Polish patients. It received ethical approval from the Bioethics Committee in Poznan (Poland) (KB 108/23). The study sample comprised a randomly-selected group of adults visiting pharmacies or healthcare centres. The inclusion criteria comprised an age of at least 18 years, and the ability to read the presented materials. No history of chronic disease including HTN was necessary. The study was performed over four months from February 1 to May 31, 2023.

The educational materials were created according to the current summaries of product characteristics (SmPCs), guidelines and recommendations [7, 14, 15]. They were then subjected to preliminary review by an interdisciplinary team of physicians and pharmacists, and evaluated with regard to their language content. All materials and their graphic design (Canva Pro) were prepared by the authors of the paper.

The materials consisted of leaflets introducing five classes of first-line hypertensive drugs: thiazide/thiazide-like diuretics, angiotensin converting enzyme, angiotensin II receptor antagonists, calcium channel blockers and beta-blockers. The readability of the language (Polish version) was evaluated according to the Plain Language Index (PLI) available at https://logios.dev/; the tool evaluates the text with regard to simple Polish language [16].

In detail, the PLI index measures ten style features, with 10% points being available for each feature. The material exceeds the PLI threshold of ≥ 40%. However, it is important to note that the algorithm does not verify the substance of the text. The following categories were evaluated by the PLI index: (1) FORMAL (whether the language style is formal and official); (2) TERMS (two-word SPECIALIST EXPRESSIONS: noun + adjective); (3) TOP100 (most frequent words in the text); (4) DWORDS (difficult – rare and long words); (5) PRON (pronouns in the text); (6) N/V (nouns/verb ratio); (7) GRAM (inadequately-used grammatical forms); (8) ASL (sentence length); (9) SENDER (how often the sender reveals their “presence” in the text, by using the words: “me”, “mine”, “our”); (10) RECEIVER (how often the sender addresses the recipient directly, by using the words: “you”, “your”, “take”).

The material, regarding HTN treatment, was then tested in an observational trial. The participants were informed of the nature of the study and that participation was voluntary, confidential and anonymous. All gave their consent to take part. They were asked to read the material, and then complete the test (see below). The participants received an individual five-minute consultation to explain the purpose of the survey and how to complete the documentation. The knowledge test was completed twice, i.e. before being presented with the material (PRE-TEST) and after reading it (POST-TEST). In addition, at the end of the study, the participant completed a questionnaire about their overall rating of the material and various aspects, such as its presentation and readability. Although the participants were not given a time limit to familiarize themselves with the material or to complete the questionnaire, the researcher recorded the time spent reading the leaflet, as well as any comments about the overall comprehensibility of the proposed material. The participants familiarized themselves with the educational material and completed all the questionnaires at the survey location, i.e. community pharmacies or healthcare centres. Only fully-completed questionnaires were included in the analysis.

### Instruments

a) The educational material comprised several paragraphs concerning the following topics: the criteria for the diagnosis of HTN, blood pressure monitoring, HTN therapy management. It also focussed on a particular therapeutic agent, its mechanism of action, indications, side effects, special warnings, information for special groups of patients, such as pregnant or nursing mothers, as well as the proper usage of the medication. The material was designed with the possibility of personalization, as it provided a space to complete the patient name, medication brand name and dosage according to general practitioner (GP) recommendations, as well as additional pharmacist information. The final version of the material, including the preliminary language check and comments from the participants, is given in Appendix B (Figures B1 − B5).

b) Specific knowledge test. This quiz included thirteen questions intended to verify that the participants understood the proper use of the hypotensive medications (thiazide/thiazide-like diuretics or angiotensin converting enzyme inhibitor) given in the material. The first six questions (questions 1 to 6) required the participants to indicate one answer out of four. The next two multiple-choice questions required them to choose from eight possible answers, with more than one answer being correct (questions 7 and 8). Finally, the next five questions were true/false statements, with the study participants indicating one of three options: *true*, *false* or *I do not know* (questions 9 to 13) (see Appendix A).

c) A questionnaire for subjective appraisal of the educational material. The tool contained three sections. The first consisted of five multiple-choice questions about age, gender, place of residence, education and history of chronic disease. The second included eight statements about the attitudes of the respondent toward the proposed educational material, including its readability, sufficiency of provided information, comprehensibility and presentation; the responses were based on a 5-point Likert scale. The respondents were also asked to subjectively rate their knowledge about hypotensive medication before and after reading the leaflet. The possible responses were arranged on a five-point scale ranging from “strongly disagree” (1 point) to “strongly agree” (5 points). The Likert Scale was proposed as it allows respondents to self-report the extent of their agreement or disagreement with a statement; such an approach might reveal subtle differences in subjects’ opinions, instead of a simple “yes” or “no” answer. The last section included two open-ended questions about participant comments concerning the educational material.

The knowledge test and the subjective appraisal of the material did not require information about the health of the respondent (quality of life, chronic disease management or well-being). However, one question enquired into any history of chronic disease, with the aim of differentiating the patients into subgroups for further analysis, i.e. subjects with history of HTN and the remaining ones.

All the materials were provided in Polish and were translated into English. The knowledge test was constructed randomly and subjectively by a seven-member panel comprising interdisciplinary academic experts with relevant expertise and experience within Pharmacy education (panels: pharmacotherapy, pharmaceutical care, drug information and communication), as well as community pharmacists with experience in patient counselling. The panel proposed a list of 25 common questions about the indications, contraindications, side effects, special warnings and proper usage of hypotensive medications; these were prepared according to their prior experience with non-adherent patients who may had misunderstood other informative materials such as patient information leaflets (PILs). Finally, this list was shortened to 13 that were included in the final version of the knowledge test. Any disagreements regarding the selection or interpretation of questions were resolved through verbal discussion until consensus was reached.

To test the questions and reduce the risk of any misunderstanding or misinterpretation, the materials and instruments were pre-tested on a sample of 10 randomly-selected respondents from the target population. Their responses were used for further clarification of the materials before the beginning of the survey and hence were not included in the subsequent analysis.

Data analysis. The results were analysed with STATISTICA 13.1 software (StatSoft Polska Sp. z o.o. 30–110 Kraków, Poland). Selected categorical socio-demographic variables were compared with the chi-square test. To identify participant attitudes toward the material, the non-parametric Kruskal–Wallis one-way analysis of variance was used to compare the mean ranks for quantitative responses (1–5 points), according to a set of covariates (e.g., history of HTN).

For each question in the knowledge test, any change in understanding about anti-hypertensive drugs associated with the learning material was assessed with the Cochrane Q-test. For this purpose, an individual correct response, given by a particular respondent in the PRE- and POST-TEST, was awarded 1 point, and an incorrect answer with 0 points. The total score achieved by a particular respondent, before and after reading the material, was presented as a percentage of possible correct answers, where 100% indicated a flawlessly completed test. The absolute difference between total scores achieved before and after reading the material was calculated, and then analysed using one-way analysis of variance (ANOVA) or Kruskal–Wallis one-way analysis of variance, with *post hoc* comparisons according to subgroups, e.g. male vs. female. The normality of the distribution of a parameter was checked with the Shapiro-Wilk test, and the homogeneity of variance with the Brown-Forsythe test. Correlation analyses were performed using the non-parametric Spearman’s test. A *P*-value of less than 0.05 was considered statistically significant.

## Results

A total of 401 patients completed the survey, i.e. with a 100% response rate; these were randomly allocated to the thiazide/thiazide-like diuretic (D) and angiotensin converting enzyme inhibitor (A) group. More detailed demographic characteristics of the study participants are presented in Table A1 (Appendix A). Of the group, 32.8% reported a history of HTN and were reading material about thiazide/thiazide-like diuretics, while 29.0% were reading about angiotensin-converting enzyme (*P* > 0.05, difference between groups). None of the other covariates, including age, gender and education, differentiated the subgroups.

### Language readability

After adjustment, the readability, grammar and structure of the proposed educational material were found to be suitable for specialist texts (i.e. ≥40% PLI) (Table A2, Appendix A).

### The knowledge test

The median time for learning the leaflet was six minutes (IQR; 6, 8), the knowledge test was completed within less than 10 min (pre-and post-test), and the approximate time spent on the survey was 20 min. The percentage scores obtained in POST-TEST and PRE-TEST are presented in Table A3 (Appendix A). A significantly higher percentage of correct answers was found in the POST-TEST than the PRE-TEST (*P* < 0.001) with the exception of Question 6.

The median score achieved before reading the leaflet was 46.8% (IQR; 31.2, 60.0) for thiazide/thiazide-like diuretics, and 43.7% (31.2, 56.2) for angiotensin-converting enzyme inhibitors; this value increased significantly in the POST-TEST to 86.7% (73.3, 93.3, *P* < 0.001 – vs. PRE-TEST) and to 75.0% (62.5, 81.2, *P* < 0.001 vs. PRE-TEST).

**Fig. 2:**
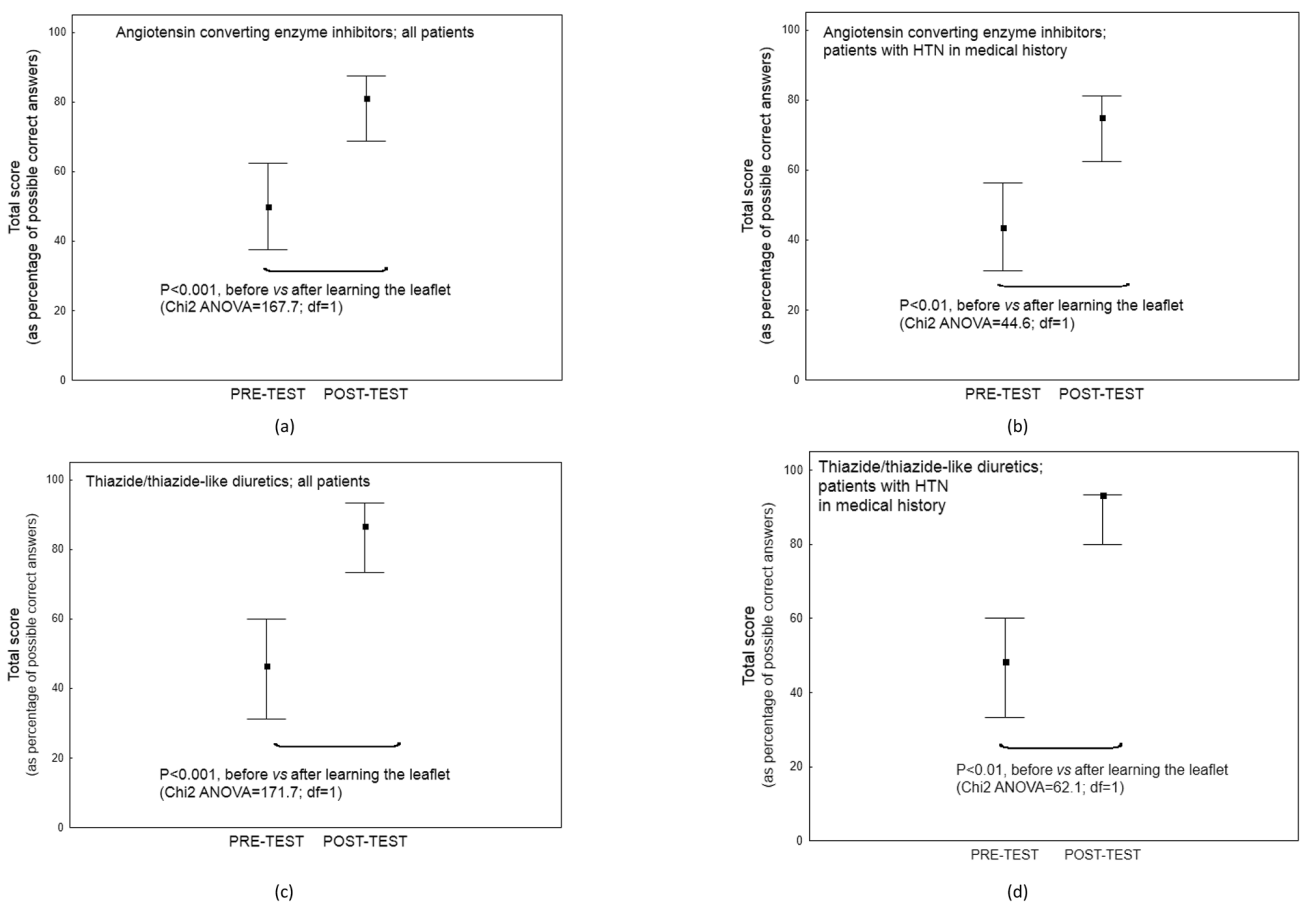
The results of knowledge test before and after learning the materials (median and 25th − 75th quartile)

**Fig. 3:**
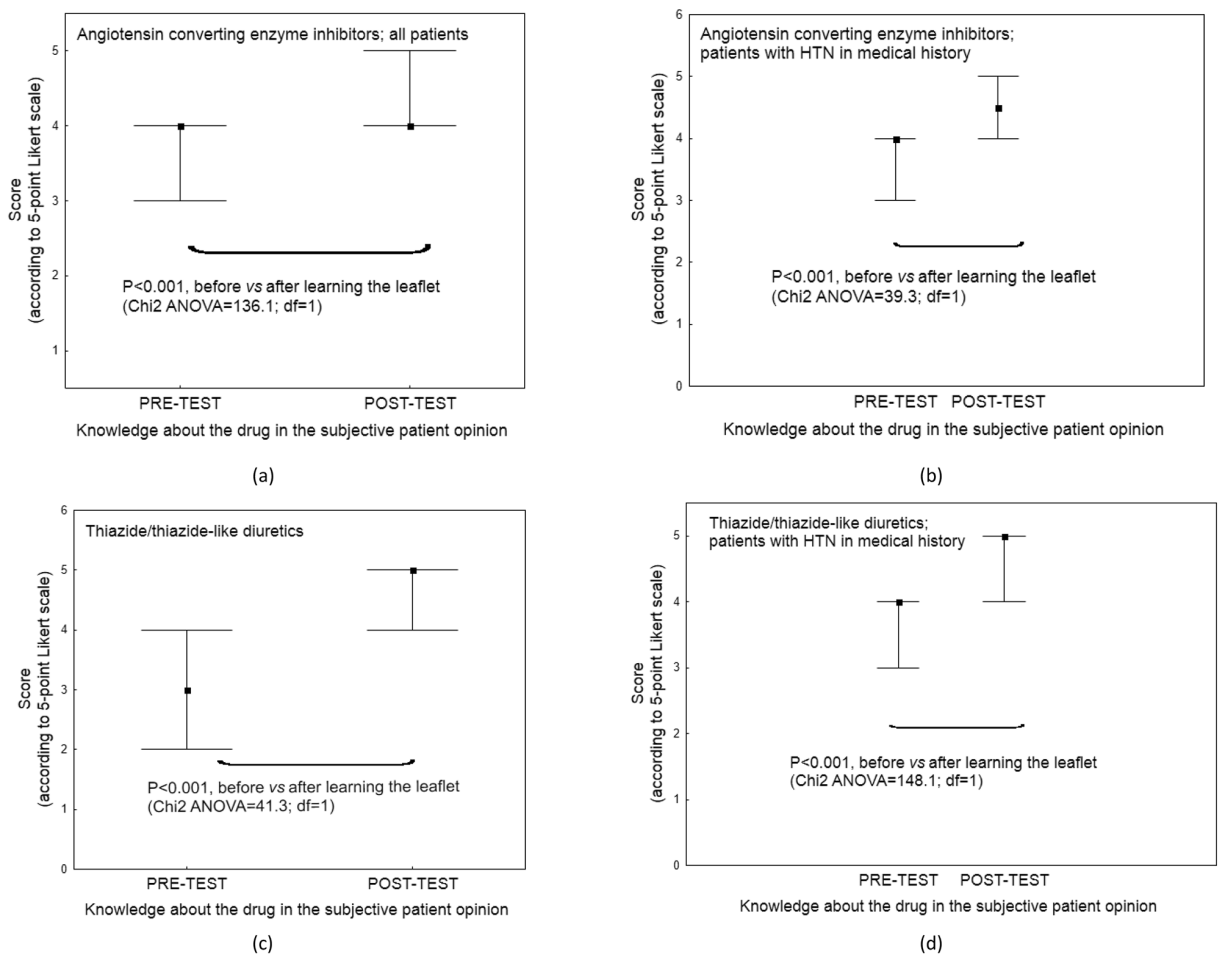
The knowledge about a drug in the subjective opinion of respondents (median and 25th − 75th quartile) (**a** – **d**)

Patients noted a subjective improvement in coping with the hypotensive drug (Fig. 2). No significant difference in basic knowledge about hypotensive drugs i.e., the PRE-TEST value, was found between the patients with a history of HTN and those without (*P* > 0.05) (Table A3, Appendix A). Both hypertensive and normotensive respondents indicated that the educational material improved their knowledge about hypotensive medication (Fig. 3). No significant difference in the increase in total test score, and hence understanding, was found between hypertensive and normotensive patients (*P* > 0.05). The final outcome did not appear to be influenced by any other sociodemographic covariates (*P* > 0.05) (Table A4, Appendix A).

The patients with a history of HTN required a significantly longer median time to read the leaflet (*P* < 0.05). In both groups, i.e., thiazide/thiazide-like diuretics, and angiotensin converting enzyme inhibitor, participants with only primary or vocational education needed a significantly longer time (*P* < 0.001).

### The educational material – appraisal

**Fig. 4:**
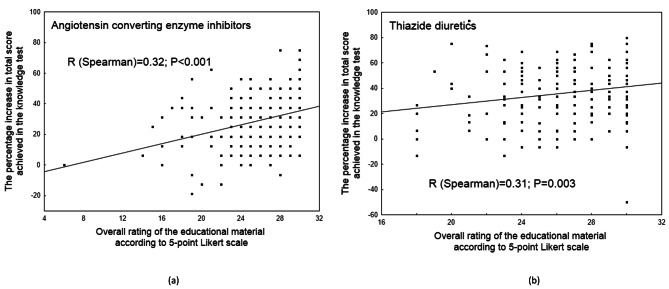
Linear regression for the percentage increase in total score achieved in the knowledge test as a function of the overall rating of the educational material − given by each individual respondent (**a** – **b**)

The participants found the educational material to be clear and well-presented, and provide sufficient information about medications for HTN. The overall rating of the educational material according to a 5-point Likert scale is given in Table A5 (Appendix A). The final outcome was not differentiated by sociodemographic covariates (*P* > 0.05) (Table A6, Appendix A). A significant correlation (*P* < 0.01) was found between the total score awarded by a particular respondent and the percentage improvement in leaflet understanding (Fig. 4).

## Discussion

Our study presents a pioneering example of educational materials that community pharmacists can use as an additional tool when performing NMS. The role of community pharmacists has evolved for years in the US and some European countries, and now exceeds the traditional dispensing of medicines; however, in Poland, the process of implementing patient-centred approaches including structured counselling or medicine reviews has generally been very slow. For example, an NMS was only officially introduced in January 2023 [4]. Pharmacists attribute the reluctance to implement the service to the low availability of supporting materials, together with the need to develop relationships with GPs and the lack of payment structure.

Our findings indicate that the prepared leaflets successfully informed patients about their medications, with an approximately 35% higher score in the POST-TEST. Such evidence-based and critically-evaluated educational materials can support medication adherence by helping the patient make the decision to take medicines, and empower management of chronic therapy, as demonstrated in other studies on patients with asthma, diabetes or rheumatoid disorders [17–19]. As adherence is best ensured by multifaceted interventions, patient education should be accompanied by other approaches e.g., simplification of treatment regimens, and communication between patients and their health care professionals, follow-up and monitoring [10].

Each registered medication provides an information insert in its package, i.e. the PIL, which includes relevant data for use. Worryingly, only a third of patients fully understand the leaflet, while less than half usually read it [20], thus raising concerns of impaired disease management and reduced medication adherence. The materials proposed in our survey have been designed to support education about new medication, and feature simplified language and content. The latter covers various topics, including the basic and crucial aspects of blood pressure monitoring and HTN therapy management, as well as basic data on the product such as dosage, mechanism of action, indications, side effects, special warnings, information for particular groups of patients including pregnant or nursing mothers, special warnings and precautions.

Instead of sections introducing recommended dose and dosage schedules, the material provides a space that can be completed by the pharmacist according to the GP’s recommendations to indicate the dosage for an individual user. Many patients indicate that anxiety arising from reading about potential side effects can influence their withdrawal from treatment or reduce adherence [20]. Hence, the section introducing medication side effects has been shortened, with the assumption that the pharmacist can use additional materials for healthcare professionals e.g. SmPC, to provide a more personalised service when counselling the patient.

The first finding is that the material significantly improved patient knowledge about the hypotensive medication. This was true for all items except question no 6, introducing the situations where the patient should stop taking the drug immediately. Interestingly, the respondents were more likely to indicate that the patient must not discontinue the medication under any circumstances (60%), rather than discontinue when experiencing any of the noted serious side effects (swelling of the face, lips, mouth, tongue or throat, difficulty in breathing). In addition, the percentage of correct answers did not increase significantly after learning the material, emphasizing the need to discuss this aspect of drug safety with patients rather than provide written materials.

This was also true regarding awareness of the most frequent adverse effects of hypotensive medication. Patient knowledge about the side effects of hypotensive medication was relatively poor – even after reading the leaflet (≈ 50% of correct answers in POST-TEST), compared with the other sections. For example, asthma was regarded as the most frequent adverse event for ACEI (33%), probably due to mistakenly associating dry cough with asthma; this item could be explained more clearly by the health care professional. In addition, 10% indicated that oedema (swelling) was an adverse event for the thiazide/thiazide-like diuretics, which might indicate a misunderstanding in the mechanism of action: diuretics increase excretion of water and sodium by the kidneys, which can reduce oedema and lower blood pressure.

Although the respondents did not require any history of HTN to take part in the current survey, the participants were sorted into hypertensive and normotensive subgroups for analysis. Among the former, the median time for hypotensive therapy (since the diagnosis) was calculated as eight years. Interestingly, the comparative analysis did not reveal any significant differences in basic knowledge, i.e. before learning the material, regarding the rationale for hypotensive therapy, proper usage, overdosage or precautions for the hypotensive medicine. The analysis did not include any questions regarding mechanisms of action or side effects, which were specific to individual therapeutic groups, but focused on the basic rationale for anti-hypertensive therapy and general rules for its proper management.

The HTN patients only obtained higher scores when they were asked about the proper drug supply (swallowing with at least half a glass of water, question no 10). Surprisingly, they provided incorrect responses to question 1, i.e. that the hypotensive drug “sometimes lowers and sometimes decreases BP” (question no 1) (11%), question 2, i.e. what to do when missing a dose (38.4%), or question 6, when experiencing serious side effects (65%) (see above). In addition, some respondents would give their drug to the other person experiencing the same symptoms (question 12) (7.1%). These gaps in patient knowledge about medication can result in poor medication adherence, which is a well-recognized contributing factor of uncontrolled hypertension [21]. This is supported by studies indicating less than 50% of adherence to treatment, expressed as proportion of days covered, in as many as half of the cohort of patients [22]. This concerns especially first-line pharmacotherapy agents like thiazide/thiazide-like diuretics and spironolactone [23], where the most common reasons for withdrawal could be adverse effects of medication or impaired quality of life [24].

It is important to emphasise that our survey did not aim to assess the direct impact of the designed educational material on medication adherence. Nevertheless, recent data from hypertensive patients provide a good evidence that higher health literacy can be associated with better medication adherence, and even blood pressure control [25]. As such, regular patient education and counselling may address the gaps in knowledge indicated among the HTN patients in the present study, thus assisting them in making informed decisions regarding their care, and improving adherence. In general, this also highlights the need to involve Polish pharmacists in patient-centred activities.

The overall appraisal of the proposed material (amount of information, its presentation, readability) was positive. The language was checked and revised for better clarity and readability before the observational study. The participants had an opportunity to give their feedback regarding overall comprehensibility of the material. Some concerns regarded the terminology, and terms perceived as too technical were reworded; for example, ‘electrolytes’, were replaced by ‘potassium level’ or ‘sodium level’ as appropriate; the term for gout was explained by additional colloquial terms for this disease in Polish.

Interestingly, while overall appraisal was not influenced by covariates such as age, gender, education or history of HTN, respondents who gave higher ratings for the material tended to obtain better scores in the knowledge test. This result is in line with previous studies, where the participants claimed to have never ‘bothered’ to read the information included in the PILs for anti-hypertensive or diabetic drugs; their criticisms concerned the limited usefulness of the PIL due to poor readability (e.g., small font size), legibility, length, design, appropriacy of the content, and difficulty of technical language [19]. This might explain, at least partially, poor disease insight and non-adherence to HTN management [26]. The current observations also underline the need for comprehensive and “patient-friendly” educational leaflets as part of a wide spectrum of intervention tools aimed at counselling chronically-ill subjects.

The designed educational material was then used to train licensed Polish pharmacists. The six-hour online courses were performed by the present authors in cooperation with four Polish medical universities (Poznan, Lodz, Krakow, Lublin) and the Supreme Pharmaceutical Chamber and Polish Pharmaceutical Society. A total of 4000 pharmacists completed the certified course to perform the New Medicine Service. The course was aimed to (a) introduce the New Medicine Service as a form of pharmaceutical consultation; (b) present the standard operating procedures (SOP), together with the algorithm of service and proposed documentation; (c) present educational strategies for improving patient knowledge about diseases and treatment, including anti-hypertensive medications. The participants reported that they found the proposed material useful for effectively educating hypertensive patients about their disease and pharmacotherapy. Further studies have been planned to design and evaluate other materials that can be used by pharmacists to inform patients.

### Limitations

The study has some limitations. The detailed analysis concerned only two subgroups of patients: those evaluating educational material for ACEI and for thiazide/thiazide-like diuretics. The materials concerning the remaining groups of antihypertensive agents (i.e., beta-blockers, calcium receptor antagonists and angiotensin type 2 receptor antagonist) were designed based on a similar scheme, including the language and layout. Also, many of the respondents had completed a high level of education (up to 42% of subjects); however, our findings do not indicate that education, or other demographic factors, influenced the final outcome in the survey.

## Conclusions

The role of the pharmacist in caring for hypertensive patient encompasses medication management, education and counselling. This may concern self-monitoring of blood pressure, dietary modification, regular physical activity, as well as adherence to pharmacological treatment recommendations. Provision of adequate, appropriate and effective written educational materials, when integrated with other educational tool and interventions, and in cooperation with health care professionals, might increase the chance of successful disease management.

The materials designed for the present study successfully improved patient knowledge about anti-hypertensive medications, and were positively appraised with regard to their readability, sufficiency of provided information, comprehensibility and presentation. Polish and English language versions of the materials can be downloaded from the national website to support patient-centred activities performed by community pharmacists. However, our findings also indicate that patients with a history of HTN may have gaps in their knowledge about the disease and the treatment, indicating the need for more effective involvement by Polish pharmacists. Particular effort should be put into educating and counselling the patient about the safety profile of anti-hypertensive medications, and presenting management strategies for situations where they might experience any serious and/or life-threatening side effects. In Polish community pharmacies, these activities might be performed during drug dispensing, or as a part of an NMS or other pharmaceutical care services outside the dispatch room. However, the latter needs further financial support by the Polish government to make it a universal service.

### Electronic supplementary material

Below is the link to the electronic supplementary material.

Supplementary Material 1

Supplementary Material 2

## Data Availability

Data generated or analysed during this study are included in this published article and its supplementary information files.
