# Supplementary material for: An evaluation of written materials for supporting hypertensive patient education and counselling when performing a new medicine service in Poland
Source: BMC Med Educ. 2024 May 10;24:521. doi: 10.1186/s12909-024-05523-x (PMC11088063; doi:10.1186/s12909-024-05523-x)
Supplement: Supplementary file 1 — Supplementary Material 1 [file 12909_2024_5523_MOESM1_ESM.docx]

**Appendix. The knowledge test with correct answer(s) marked**

Thiazide/thiazide-like diuretic

(1) The drug is intended to (mark the right answer): 🗹 lower blood pressure (BP); sometimes lower and sometimes decrease BP; increase BP; I do not know

(2) If you forget to take your new medicine as prescribed (mark the right answer):  call an emergency;  stop taking your drug and see the doctor for a different medication; 🗹 take the next dose at the usual time;  I do not know

(3) I can double the drug dose (than prescribed by the doctor) (mark the right answer):  if the blood pressure, measured at home or at the pharmacy, is higher than usual;  if my well-being worsens; 🗹 I cannot take more medicine than prescribed, myself;  I do not know

(4) When you are taking the drug (mark the right answer):  I can drive all the time, as the drug does not affect the ability to drive a car safely;  driving a car is not allowed at all; 🗹 when I feel drowsy or dizzy, driving a car is not recommended;  I do not know

(5) If you take more medicine than prescribed (mark the right answer):  blood pressure will rise significantly; 🗹 blood pressure will drop significantly ;  blood pressure will not change;  I do not know

(6) I can stop taking the drug yourself (mark the right answer):  if my blood pressure is normal; 🗹 if I experience any of the following side effects that can be serious e.g., swelling of the throat and difficulty in swallowing;  I can never stop taking the drug on my own;  I do not know

(7) Please indicate the advantage(s) of the drug:  lowers cholesterol; 🗹 maintains normal BP;  has antiviral effect;  reduces hear loss;  helps to increase the supply of blood, food and oxygen to your heart, and reduces chest pain; 🗹 reduces oedema;  I do not know

(8) Please indicate common drug adverse reaction(s):  cough;  oedema;  exacerbates psoriasis; 🗹 exacerbates gout symptoms;  exacerbates asthma symptoms; 🗹 affects laboratory test results;  constipation;  I do not know

(9) Some other drugs may be affected by the medication 🗹 TRUE;  FALSE;  I do not know

(10) The tablet should be swallowed whole with at least half a glass of water: 🗹 TRUE;  FALSE;  I do not know

(11) The drug may cause adverse reactions: 🗹 TRUE;  FALSE;  I do not know

(12) I can pass the drug to others, if their illness is the same as mine:  TRUE; 🗹 FALSE;  I do not know

(13) In case of positive pregnancy test result, the drug should be discontinued immediately without contacting a doctor:  TRUE; 🗹 FALSE;  I do not know

Angiotensin converting enzyme inhibitor

(1) The drug is intended to (mark the right answer): 🗹 lower blood pressure (BP); sometimes lower and sometimes decrease BP; increase BP; I do not know

(2) If you forget to take your new medicine as prescribed (mark the right answer):  call an emergency;  stop taking your drug and see the doctor for a different medication; 🗹 take the next dose at the usual time;  I do not know

(3) I can double the drug dose (than prescribed by the doctor) (mark the right answer):  if the blood pressure, measured at home or at the pharmacy, is higher than usual;  if my well-being worsens; 🗹 I cannot take more medicine than prescribed, myself;  I do not know

(4) When you are taking the drug (mark the right answer):  I can drive all the time, as the drug does not affect the ability to drive a car safely;  driving a car is not allowed at all; 🗹 when I feel drowsy or dizzy, driving a car is not recommended;  I do not know

(5) If you take more medicine than prescribed (mark the right answer):  blood pressure will rise significantly; 🗹 blood pressure will drop significantly ;  blood pressure will not change;  I do not know

(6) I can stop taking the drug yourself (mark the right answer):  if my blood pressure is normal; 🗹 if I experience any of the following side effects that can be serious e.g., swelling of the throat and difficulty in swallowing;  I can never stop taking the drug on my own;  I do not know

(7) Please indicate the advantage(s) of the drug:  lowers cholesterol; 🗹 maintains normal BP;  has an antiviral effect;  reduces hear loss; 🗹 helps to increase the supply of blood, food and oxygen to your heart, and reduces chest pain;  reduces oedema;  I do not know

(8) Please indicate common drug adverse reaction(s): 🗹 cough;  oedema;  exacerbates psoriasis;  exacerbates gout symptoms;  exacerbates asthma symptoms; 🗹 affects laboratory test results;  constipation;  I do not know

(9) Some other drugs may be affected by the medication 🗹 TRUE;  FALSE;  I do not know

(10) The tablet should be swallowed whole with at least half a glass of water: 🗹 TRUE;  FALSE;  I do not know

(11) The drug may cause adverse reactions: 🗹 TRUE;  FALSE;  I do not know

(12) I can pass the drug to others, if their illness is the same as mine:  TRUE; 🗹 FALSE;  I do not know

(13) In case of positive pregnancy test result, the drug should be discontinued immediately without contacting a doctor:  TRUE; 🗹 FALSE;  I do not know

**Table A1.** Demographic Characteristics of Respondents (N=401).

| Number of participants  Thiazide/thiazide-like diuretic (D)  Angiotensin converting enzyme inhibitor (A) | 201 (50.1%)  200 (49.9%) |
| --- | --- |
| Age (median and 25^th^ − 75^th^ quartile) | 52 (42, 66)^D^; 54 (40, 67)^A^ (NS^1^) |
| Female (%) . | 123 (61.2%)^D^; 131 (65.5%)^A^ (NS^2^) |
| Place of origin (%)  Village  City of <50,000 inhabitants  City of > 500,000 inhabitants | 2 (1.0%)^D^  9 (4.5%)^D^  189 (94.0%)^D^; 200 (100%)^A^ |
| Education (%)  Primary  Vocational  Secondary  Higher | 9 (4.5%)^D^; 8 (4.0%)^A^  54 (26.9%)^D^; 35 (17.5%)^A^  74 (36.8%)^D^; 73 (36.5%)^A^  64 (31.7%)^D^; 84 (42.0%)^A^ |
| Chronic diseases  Hypertension  Asthma  Diabetes  Others | 66 (32.8%)^D^; 58 (29.0%)^A^ (NS^2^)  16 (8.0%)^D^; 14 (7.0%)^A^ (NS^2^)  25 (12.4%)^D^; 22 (11.0%)^A^ (NS^2^)  28 (13.9%)^D^; 33 (16.5%)^A^ |
| Participants with hypertension in history  Age (median and 25^th^ − 75^th^ quartile)  Female (%)  Duration of the disease (years) | 62.5 (55, 70)^D^; 62.5 (54, 71)^A^ (NS^1^)  39 (59.1%)^D^; 33 (56.9%)^A^ (NS^2^)  7 (5, 12)^D^; 8 (6, 12)^A^ (NS^1^) |

NS – P>0.05 for comparison between thiazide/thiazide-like diuretic (D) and angiotensin converting enzyme inhibitor (A) group; ^1^ – according to The Mann-Whitney U test; ^2^ – according to Chi square Pearson’s test.

**Table A2.** The verification of language accessibility according to Plain Language Index (PLI).

| Feature | Before adjustment | After adjustment |
| --- | --- | --- |
| FORMAL | 0.0%^D^ 0.0%^A^ | 28.0%^D^ 28.0%^A^ |
| TERMS | 30.0%^D^ 4.0%^A^ | 39.0%^D^ 43.0%^A^ |
| TOP100 | 0.0%^D^ 0.0%^A^ | 26.0%^D^ 26.0%^A^ |
| DWORDS | 44.0%^D^ 52.0%^A^ | 71.0%^D^ 76.0%^A^ |
| PRON | 0.0%^D^ 80.0%^A^ | 47.0%^D^ 47.0%^A^ |
| N/V | 0.0%^D^ 80.0%^A^ | 47.0%^D^ 47.0%^A^ |
| GRAM | 46.0%^D^ 88.0%^A^ | 64.0%^D^ 62.0%^A^ |
| ASL | 0.0%^D^ 0.0%^A^ | 33.0%^D^ 33.0%^A^ |
| SENDER | 0.0%^D^ 0.0%^A^ | 0.0%^D^ 0.0%^A^ |
| RECEIVER | 100.0%^D^ 100.0%^A^ | 92.0%^D^ 92.0%^A^ |
| TOTAL | 33.0%^D^ 40.0%^A^ | 50.0%^D^ 49.0%^A^ |

**Table A3.** Knowledge test – the results.

| Item | Question (No) | PRE-TEST (%) | POST-TEST (%) | | PRE-TEST (%) |
| --- | --- | --- | --- | --- | --- |
|  |  | All patients | | HTN^#^ | |
| Mechanism of action/effects  *P<0.0001^D, A^ | (1) | ^D^ 73.6 | ^D^ 98.1 | 78.4 | |
|  |  | ^A^ 75.5 | ^A^ 95.5 |  |  |
|  | (7) | ^D^ 29.7 | ^D^ 71.6 | - | |
|  |  | ^A^ 54.0 | ^A^ 87.0 |  |  |
|  | (9) | ^D^ 58.2 | ^D^ 99.5 | 58.4 | |
|  |  | ^A^ 75.5 | ^A^ 96.0 |  |  |
| Adverse reactions  *P<0.0001^D, A^ | (8) | ^D^ 3.5 | ^D^ 64.7 | - | |
|  |  | ^A^ 8.0 | ^A^ 38.5 |  |  |
|  | (11) | ^D^ 71.6 | ^D^ 98.5 | 85.6 | |
|  |  | ^A^ 90.0 | ^A^ 95.0 |  |  |
| Contraindications and precautions  *P<0.0001^D, A^ | (4) | ^D^ 45.3 | ^D^ 93.5 | 43.2 | |
|  |  | ^A^ 39.0 | ^A^ 83.0 |  |  |
| Overdosage  *P<0.0001^D, A^ | (5) | ^D^ 60.7 | ^D^ 92.0 | 61.6 | |
|  |  | ^A^ 61.5 | ^A^ 91.5 |  |  |
| Proper usage  *P<0.0001^D, A^ | (2) | ^D^ 59.2 | ^D^ 97.0 | 64.8 | |
|  |  | ^A^ 70.5 | ^A^ 92.5 |  |  |
|  | (3) | ^D^ 42.8 | ^D^ 95.5 | 47.2 | |
|  |  | ^A^ 59.0 | ^A^ 93.5 |  |  |
|  | (10) | ^D^ 79.1 | ^D^ 100.0 | 85.6 | |
|  |  | ^A^ 81.5 | ^A^ 98.5 |  |  |
|  | (12) | ^D^ 77.1 | ^D^ 96.0 | 77.6 | |
|  |  | ^A^ 81.5 | ^A^ 95.0 |  |  |
| Special Warnings  NS^D, A^ | (6) | ^D^ 35.8 | ^D^ 39.8 | 36.8 | |
|  |  | ^A^ 34.0 | ^A^ 41.0 |  |  |
| Pregnancy *P<0.0001^D, A^ | (13) | ^D^ 38.3 | ^D^ 54.7 | 24.1 | |
|  |  | ^A^ 19.5 | ^A^ 58.5 |  |  |

The percentage score obtained in the knowledge test (PRE-TEST and POST-POST); * − for comparison before and after learning the material (Q Cochrane); ^#^− (H=0.04; P>0.05) for comparison the total answer score (percentage of correct answers) between hypertensive (*N=124*) and remaining patients (Kruskal-Wallis test); ^D^ – educational material for thiazide/thiazide-like diuretics; ^A^ – educational material for angiotensin converting enzyme inhibitors; NS – non significant.

**Table A4.** The influence of selected covariates on the increase of knowledge about hypotensive drug due to the proposed educational material.

| Covariate | The median increase in total score (%) achieved in the knowledge test (25^th^, 75^th^ quartile) | The comparison between groups |
| --- | --- | --- |
| Age | - | R Spearman (=0.37; P>0.05)^D^  R Spearman (=-0.08; P>0.05)^A^ |
| Gender | Females: 33.33% (20.0, 50.0)^D^ ; 25.0% (18.75, 37.5)^A^  Males: 40.61% (26.67, 56.25)^D^; 31.25% (18.75, 43.75)^A^ | NS (H=3.79; P>0.05)^D^; NS (H=2.20; P>0.05)^A^ |
| Education | Higher: 37.5% (25.83, 50)^D^; 28.12% (12.5, 37.5)^A^  Secondary: 35.42% (20, 53.33)^D^; 25.0% (18.75, 37.5)^A^  Vocational: 40.0% (26.67, 53.33)^D^; 31.25% (18.75, 50.0)^A^  Primary: 40.0% (26.67, 43.75)^D^; 12.5% (9.37, 34.37)^A^ | NS (H=0.83; P>0.05)^D^; NS (H=5.60; P>0.05)^A^ |
| History of hypertension | Yes: 37.5% (26.67, 46.67)^D^; 28.12% (18.75, 43.75)^A^  No: 40.0% (20.0, 53.33)^D^; 25.0% (18.75, 37.5)^A^ | NS (H=0.41; P>0.05)^D^; NS (H=0.57; P>0.05)^A^ |

NS – non significant; ^D^ – educational material for thiazide/thiazide-like diuretics; ^A^ – educational material for angiotensin converting enzyme inhibitors

**Table A5**. The overall rating of the educational material according to 5-point Likert scale; where 1 – strongly disagree; 2 – rather disagree; 3 – undecided; 4 – rather agree; 5 – strongly agree (median and 25^th^ − 75^th^ quartile).

| Statement | All | HTN* |
| --- | --- | --- |
| I was interested in this educational material | ^D^ 5 (4, 5) | ^D^ 5 (4, 5) |
|  | ^A^ 4 (4, 5) | ^A^ 4 (4, 5) |
| The leaflet is pleasant to read (I like the presentation of the leaflet) | ^D^ 5 (4, 5) | ^D^ 5 (4, 5) |
| The leaflet is clear and makes it easy to find the information I need | ^A^ 4 (4, 5) | ^A^ 4 (4, 5) |
| The information in the leaflet is useful | ^D^ 5 (4, 5) | ^D^ 5 (4, 5) |
|  | ^A^ 4 (4, 5) | ^A^ 5 (4, 5) |
| The amount of the information provided in the leaflet is sufficient and comprehensive | ^D^ 4 (4, 5) | ^D^ 4 (4, 5) |
|  | ^A^ 4 (3, 5) | ^A^ 4 (2, 5) |
| The information in the leaflet is understandable (I understand the leaflet) | ^D^ 4 (4, 5) | ^D^ 4 (4, 5) |
|  | ^A^ 5 (4, 5) | ^A^ 5 (4, 5) |
| The overall rate | ^D^ 26 (24, 29) | ^D^ 27 (24, 29) (H=0.51; P>0.05)^#^ |
|  | ^A^ 25 (29, 29) | ^A^ 25.5 (24, 29) (H=1.05; P>0.05)^#^ |

*HTN – subgroup of patients with history of hypertension; ^#^− P>0.05 for comparison the total answer score (percentage of correct answers) between hypertensive (*N=124*) and remaining patients (Kruskal-Wallis test); ^D^ – educational material for thiazide/thiazide-like diuretics; ^A^ – educational material for angiotensin converting enzyme inhibitors.

**Table A6**. The influence of other covariates on the overall appraisal of the proposed educational material.

| Covariate | The median increase in total score (%) achieved in the knowledge test (25^th^, 75^th^ quartile) | The comparison between groups |
| --- | --- | --- |
| Age | - | R Spearman (=0.095; P>0.05)^D^  R Spearman (=-0.17; P>0.05)^A^ |
| Gender | Females: 26.0 (24.0, 29.0)^D^ ; Males: 26.0 (24.0, 29.0)^D^  Females: 25.0 (23.0, 29.0)^A^; Males: 25.0 (23.0, 28.0)^A^ | NS (H=0.29; P>0.05)^D^; NS (H=0.063; P>0.05)^A^ |
| Education | Higher: 27.0 (25.0, 29.5)^D^; Secondary: 26.0 (24.0, 29.0)^D^  Vocational: 26.0 (24.0, 29.0)^D^; Primary: 30.0 (20.0, 30.0)^D^  Higher: 27.0 (23.0, 29.0)^A^; Secondary: 25.0 (23.0, 27.0)^A^  Vocational: 25.0 (23.0, 29.0)^A^; Primary: 26.5 (25.5, 29.5)^A^ | NS (H=2.38; P>0.05)^D^;  NS (H=4.51; P>0.05)^A^ |
| History of hypertension | Yes: 27.0 (24.0, 29.0)^D^; No: 26.0 (24.0, 29.0)^D^  Yes: 25.5 (24.0, 29.0)^A^; No: 25.0 (23.0, 28.0)^A^ | NS (H=0.51; P>0.05)^D^; NS (H=1.20; P>0.05)^A^ |

NS – non significant; ^D^ – educational material for thiazide/thiazide-like diuretics; ^A^ – educational material for angiotensin converting enzyme inhibitors
