# Supplementary material for: An evaluation of written materials for supporting hypertensive patient education and counselling when performing a new medicine service in Poland
Source: BMC Med Educ. 2024 May 10;24:521. doi: 10.1186/s12909-024-05523-x (PMC11088063; doi:10.1186/s12909-024-05523-x)

Figure B1. Educational material for the patient. Angiotensin converting enzyme inhibitor.


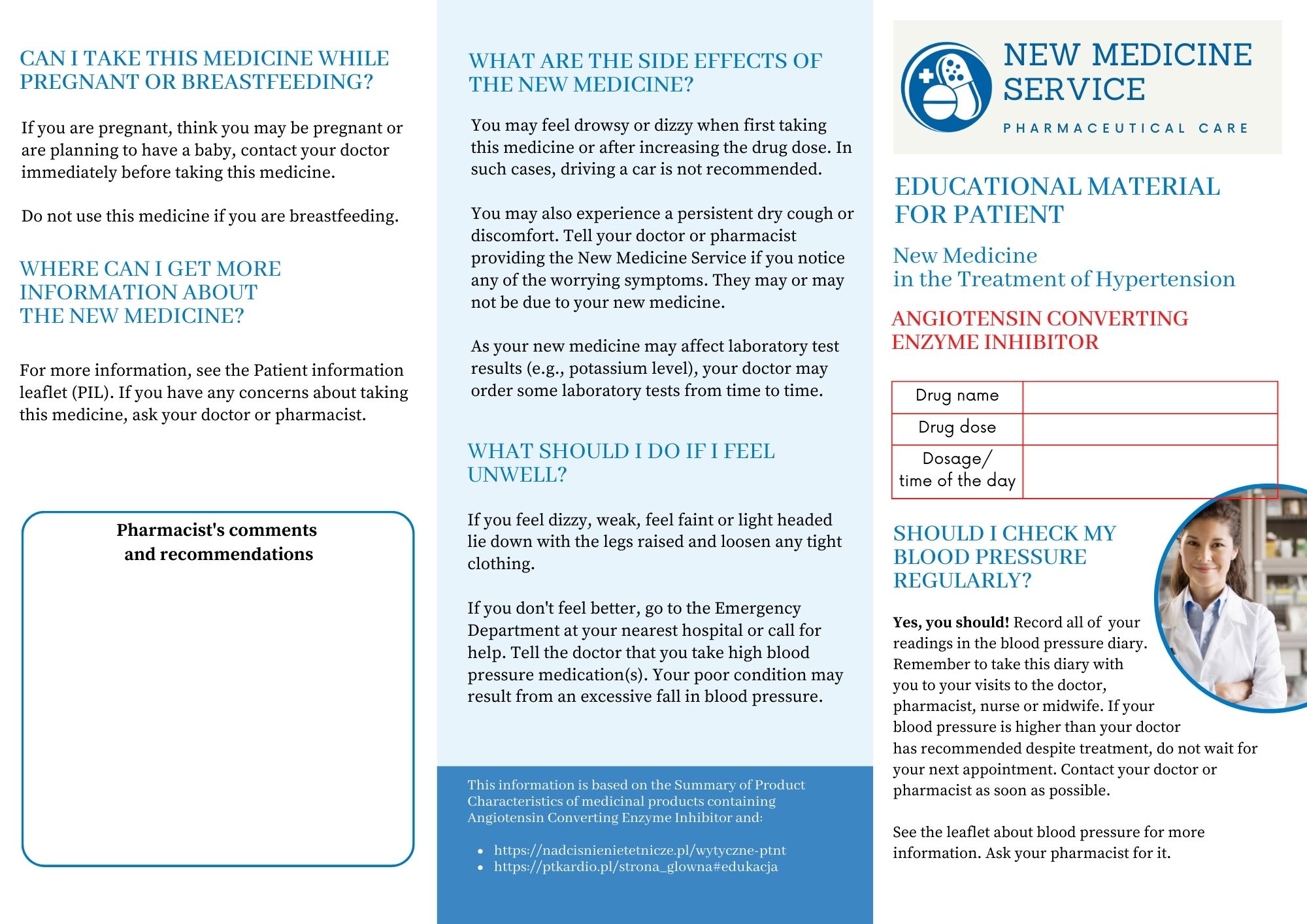


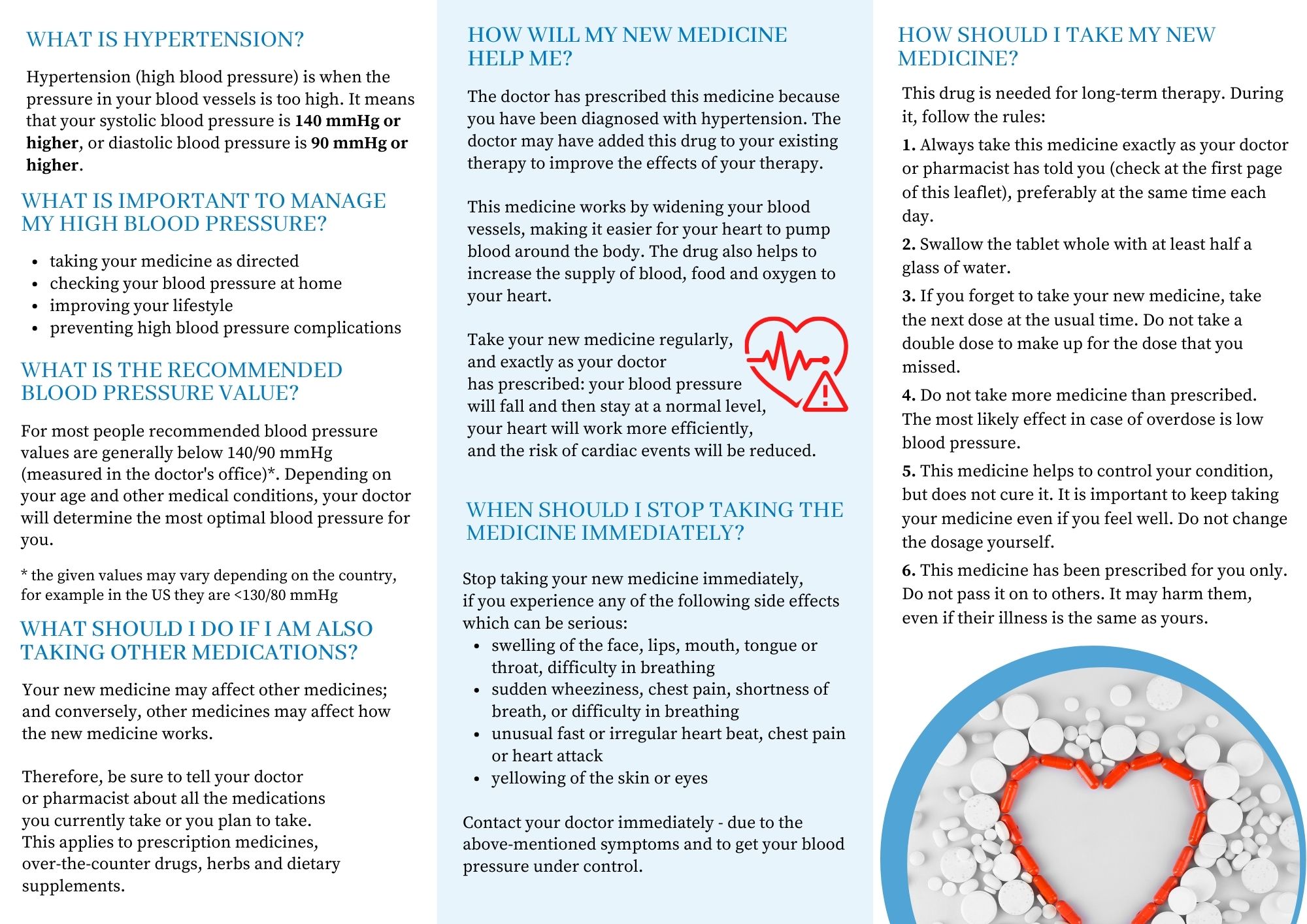


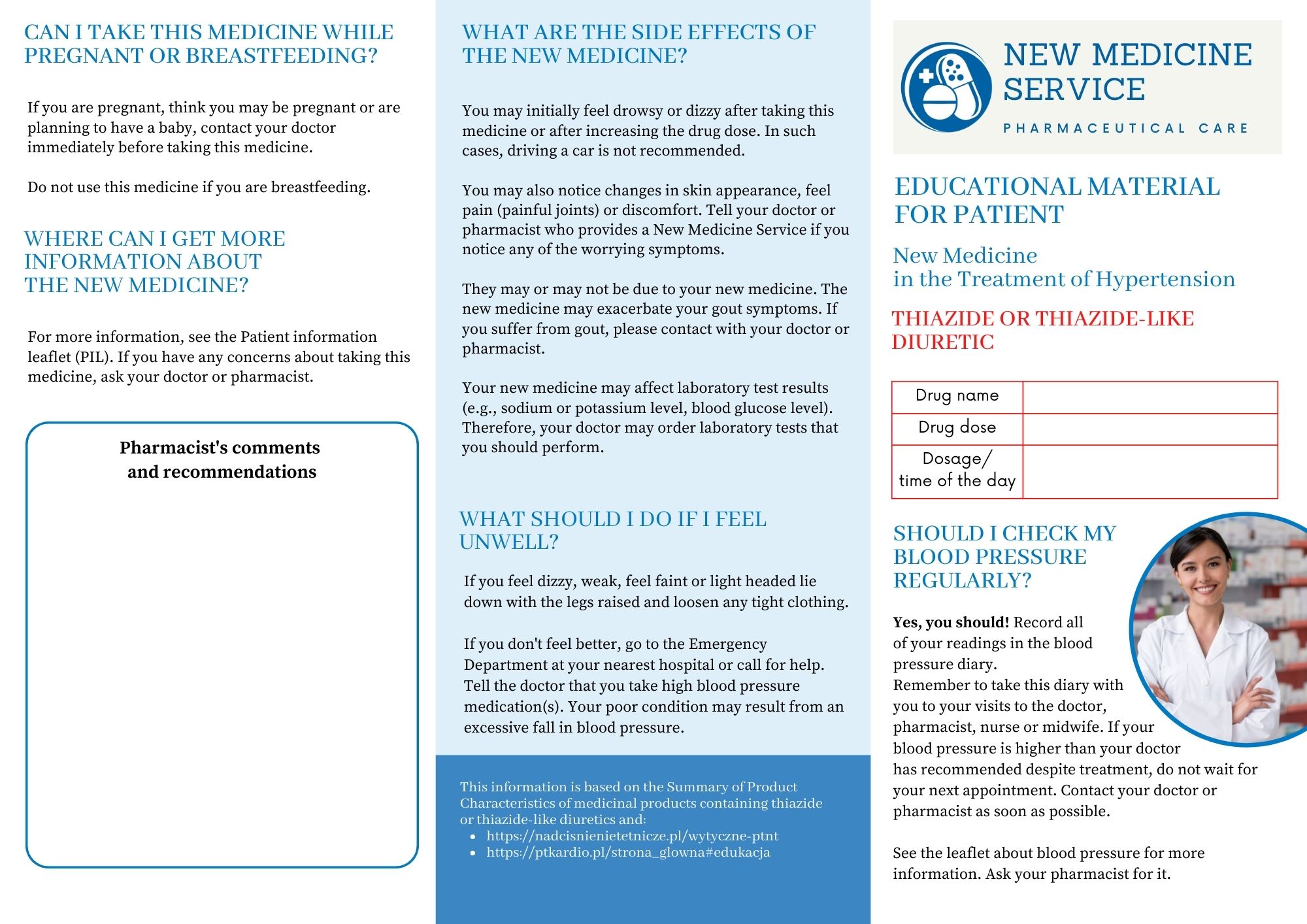
Figure B2. Educational material for the patient. Thiazide/thiazide-like diuretic.


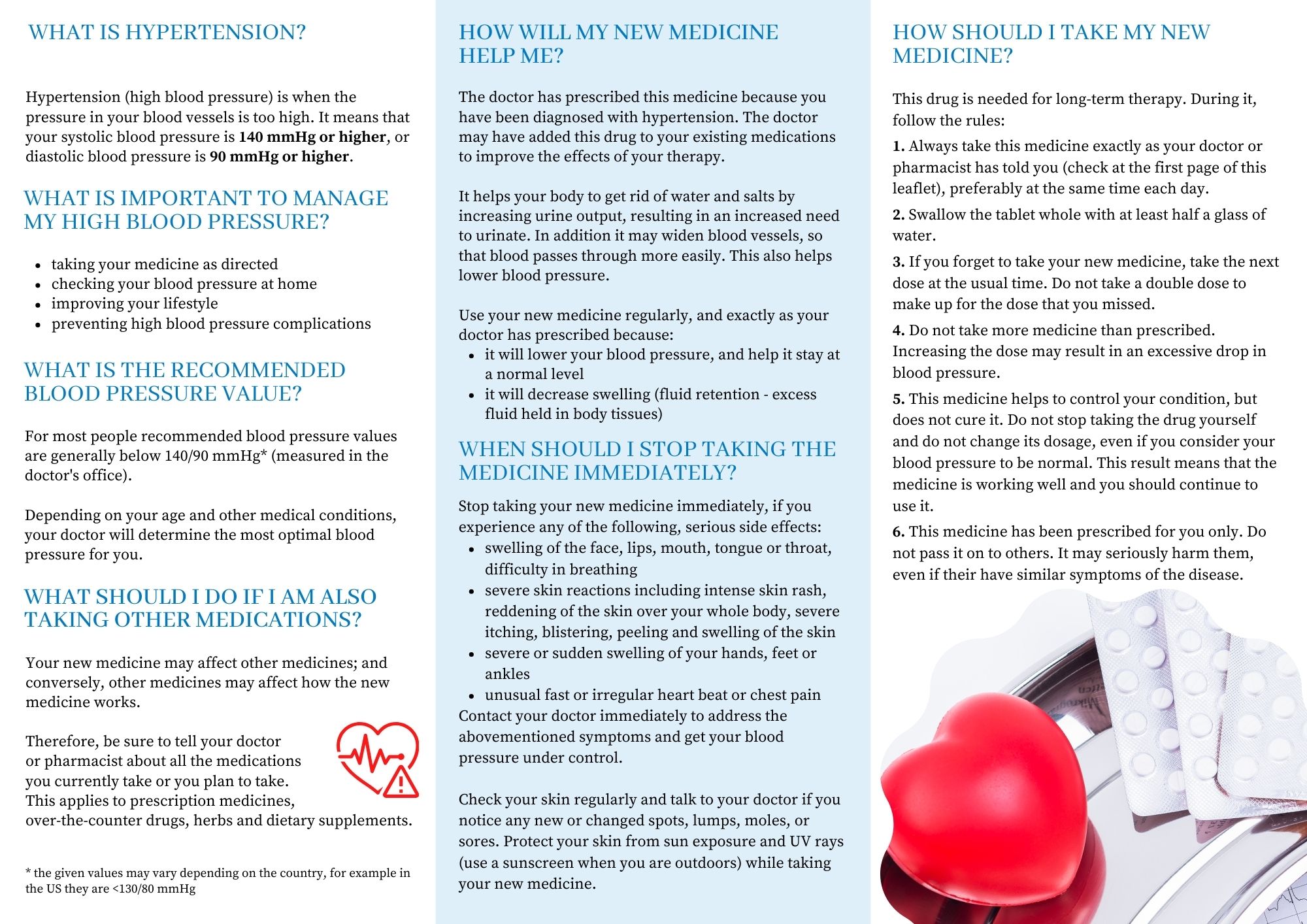


Figure B3. Educational material for the patient. Calcium channel blocker (calcium antagonist).


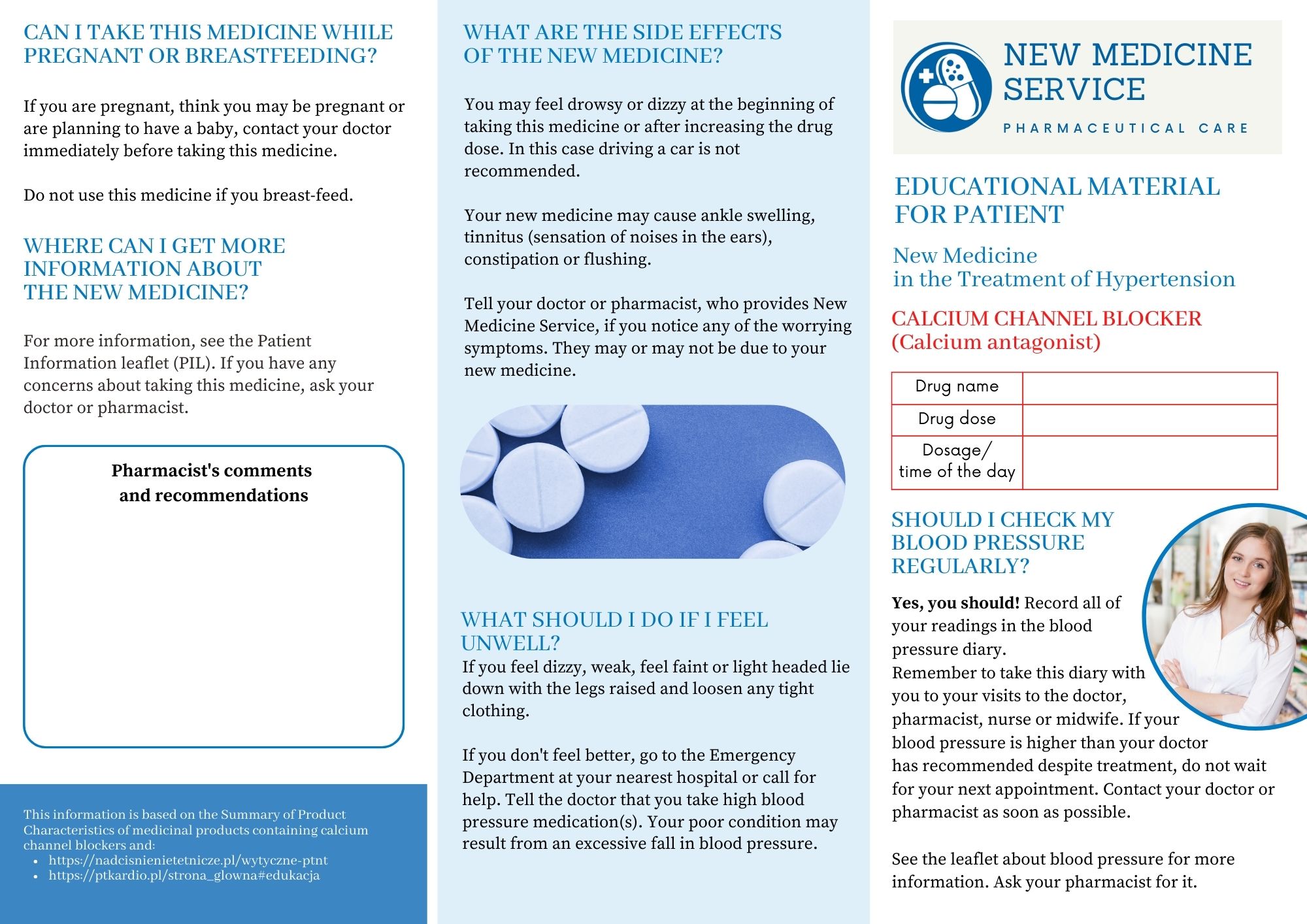


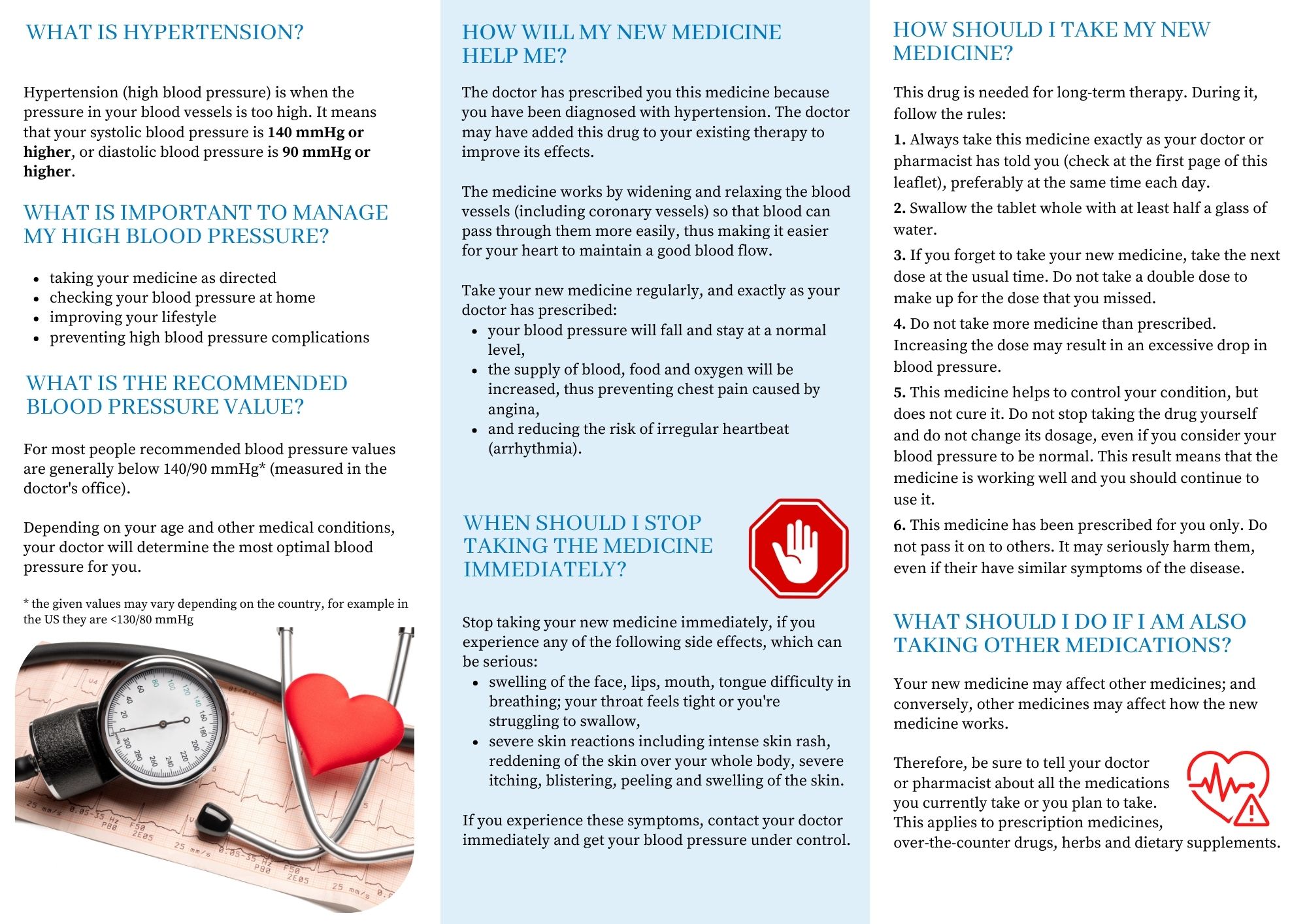


Figure B4

Figure B4. Educational material for the patient. Beta-adrenergic blocking agent (Beta-blocker).


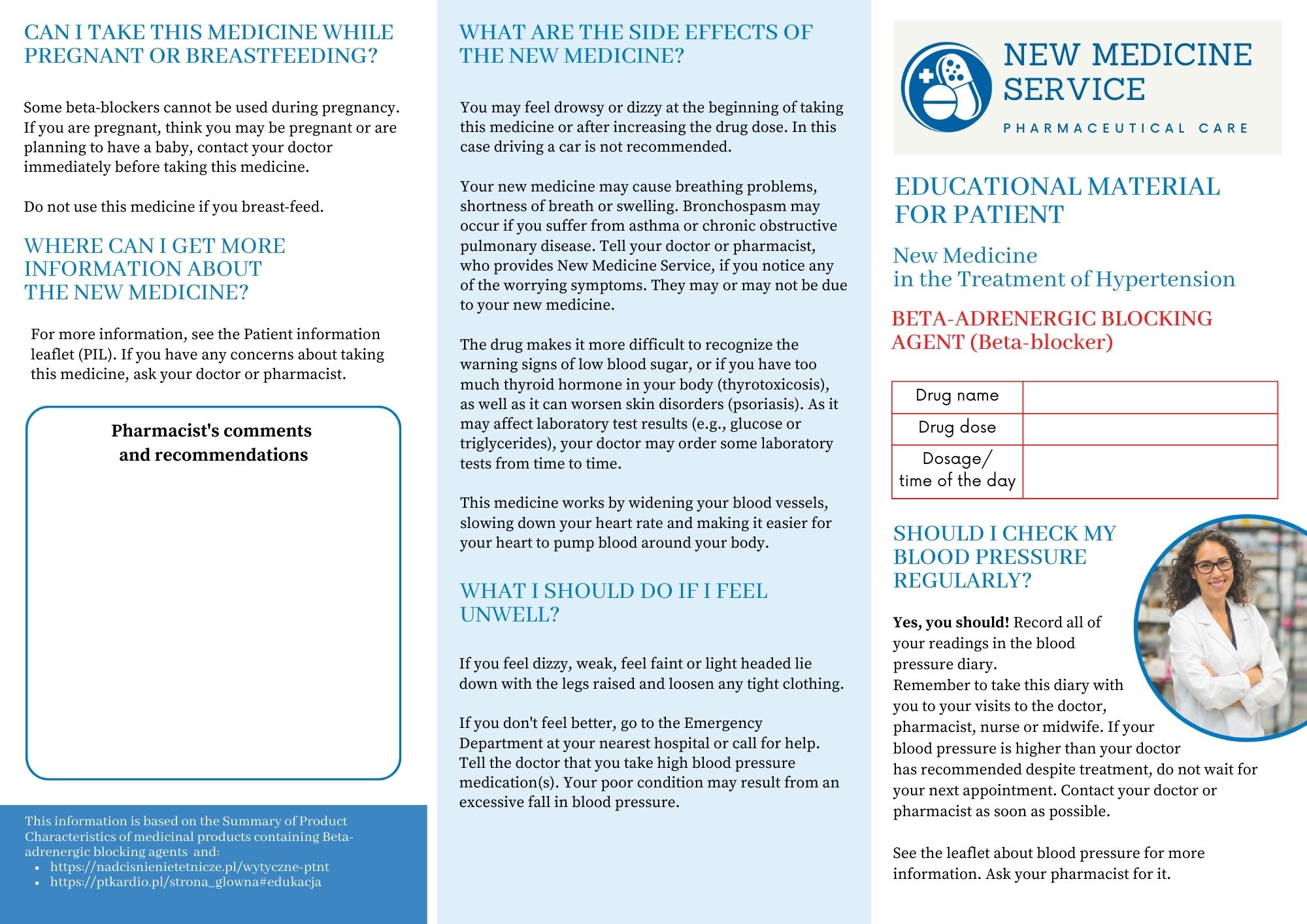


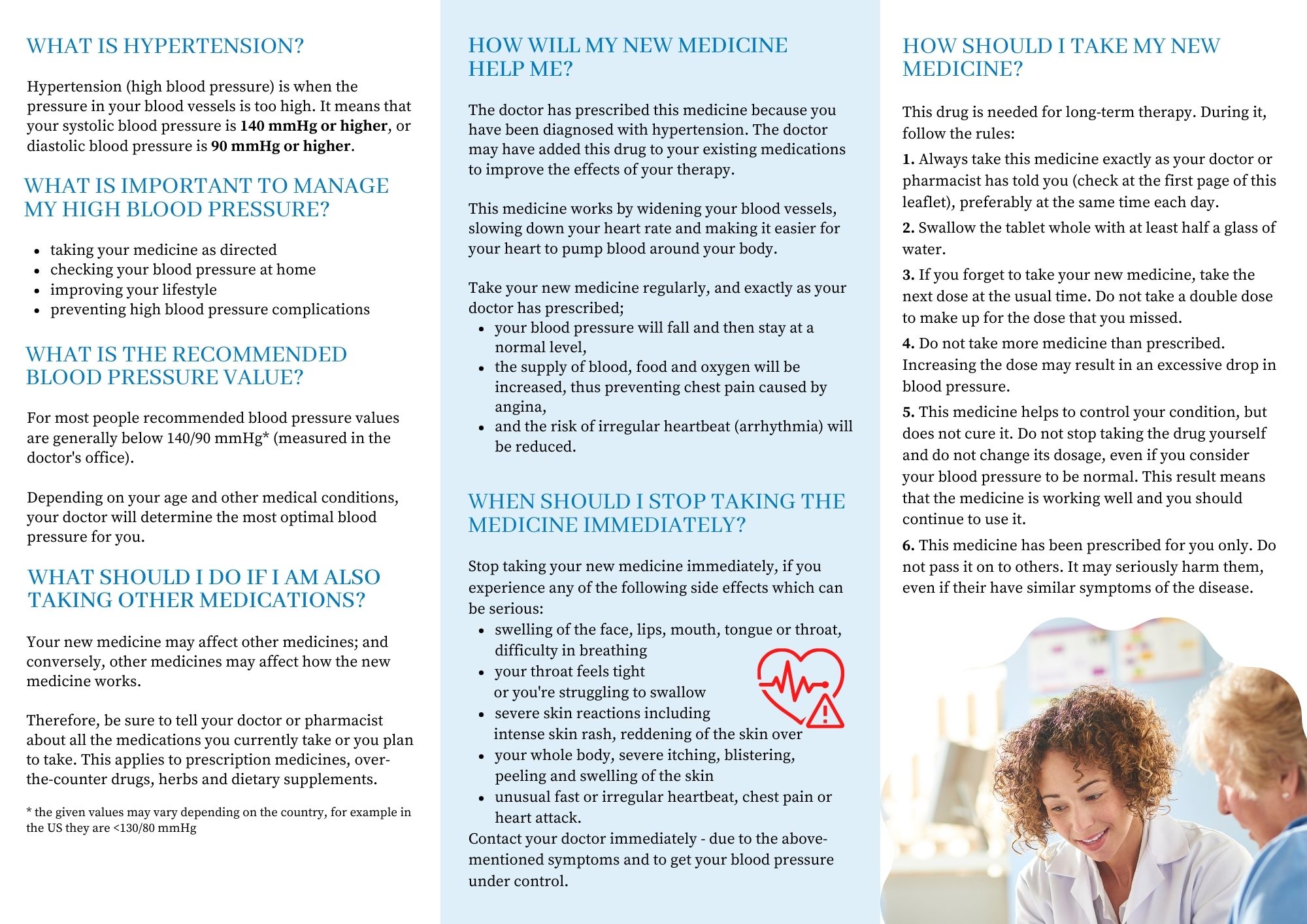


Figure B5

Figure B5. Educational material for the patient. Angiotensin II receptor antagonist.


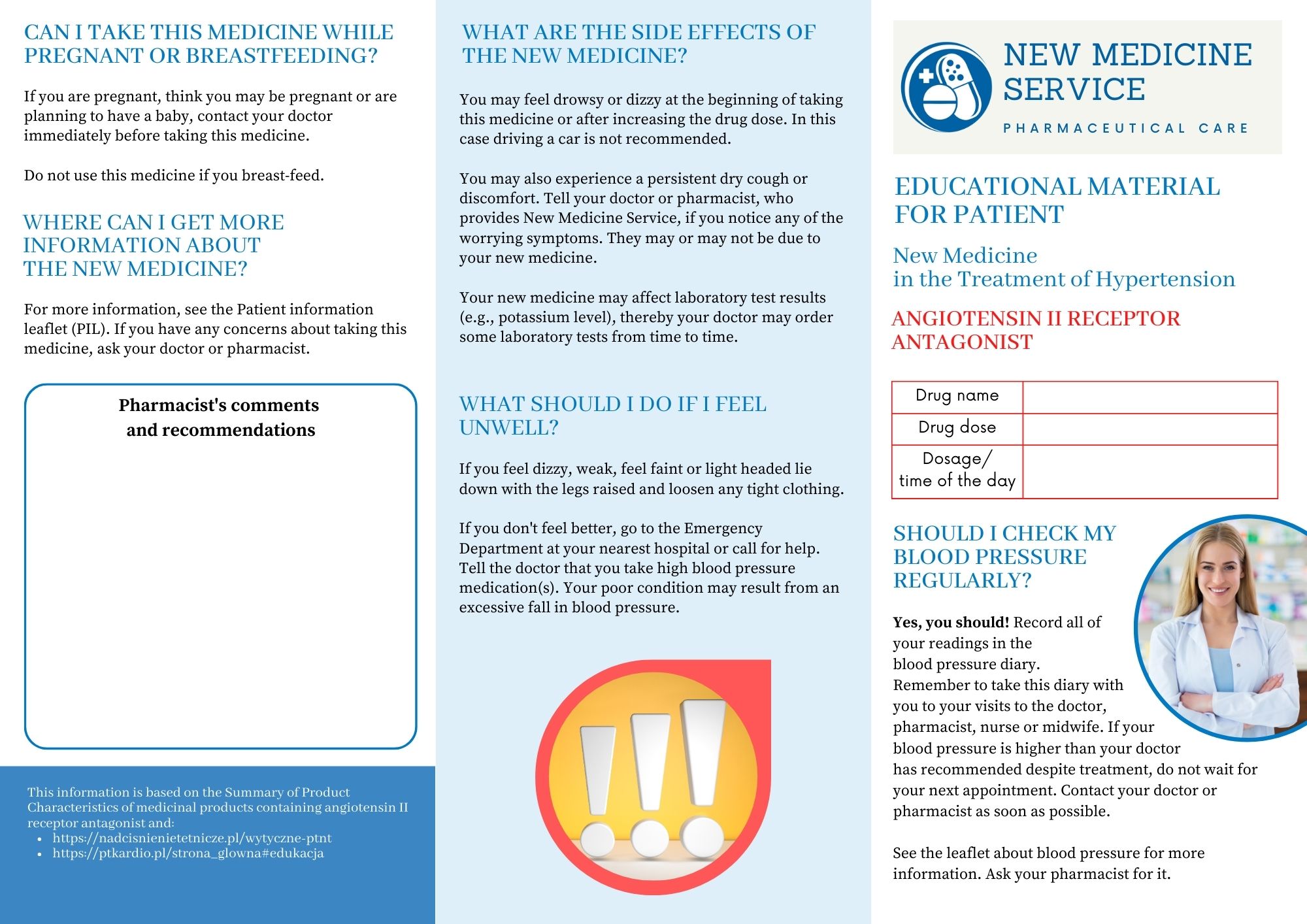


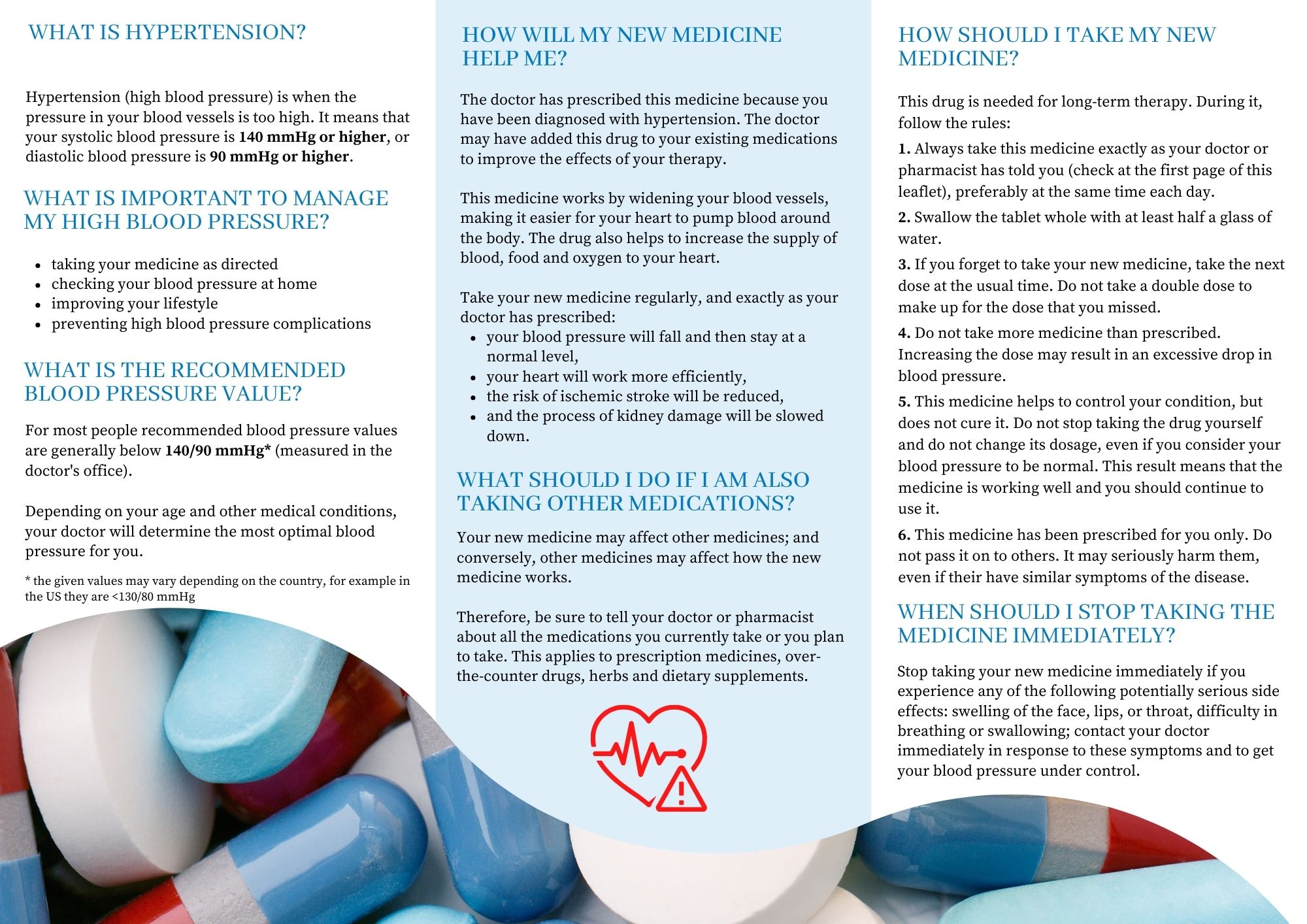

Supplement: Supplementary file 2 — Supplementary Material 2 [file 12909_2024_5523_MOESM2_ESM.docx]
